# Intellectual Function in Mexican Children Living in a Mining Area and Environmentally Exposed to Manganese

**DOI:** 10.1289/ehp.0901229

**Published:** 2010-06-01

**Authors:** Horacio Riojas-Rodríguez, Rodolfo Solís-Vivanco, Astrid Schilmann, Sergio Montes, Sandra Rodríguez, Camilo Ríos, Yaneth Rodríguez-Agudelo

**Affiliations:** 1 Dirección de Salud Ambiental, Instituto Nacional de Salud Pública, Cuernavaca, Morelos, Mexico; 2 Departamento de Neuropsicología and; 3 Departamento de Neuroquímica, Instituto Nacional de Neurología y Neurocirugía “Manuel Velasco Suárez,” Mexico City, Mexico

**Keywords:** children, environmental exposure, IQ, manganese, neurotoxicity

## Abstract

**Background:**

Excessive exposure to manganese (Mn), an essential trace element, has been shown to be neurotoxic, especially when inhaled. Few studies have examined potential effects of Mn on cognitive functions of environmentally exposed children.

**Objective:**

This study was intended to estimate environmental exposure to Mn resulting from mining and processing and to explore its association with intellectual function of school-age children.

**Methods:**

Children between 7 and 11 years of age from the Molango mining district in central Mexico (*n* = 79) and communities with similar socioeconomic conditions that were outside the mining district (*n* = 93) participated in the cross-sectional evaluation. The revised version of the Wechsler Intelligence Scale for Children adapted for the Mexican population was applied. Concentrations of Mn in blood (MnB) and hair (MnH) were used as biomarkers of exposure.

**Results:**

Exposed children had significantly higher median values for MnH (12.6 μg/g) and MnB (9.5 μg/L) than did nonexposed children (0.6 μg/g and 8.0 μg/L, respectively). MnH was inversely associated with Verbal IQ [β = −0.29; 95% confidence interval (CI), −0.51 to −0.08], Performance IQ (β = −0.08; 95% CI, −0.32 to 0.16), and Total Scale IQ (β = −0.20; 95% CI, −0.42 to 0.02). MnB was inversely but nonsignificantly associated with Total and Verbal IQ score. Age and sex significantly modified associations of MnH, with the strongest inverse associations in young girls and little evidence of associations in boys at any age. Associations with MnB did not appear to be modified by sex but appeared to be limited to younger study participants.

**Conclusions:**

The findings from this study suggest that airborne Mn environmental exposure is inversely associated with intellectual function in young school-age children.

Manganese (Mn) is an essential element. It actively participates in important enzymatic reactions for cellular function (Gwiazda et al. 2007). Nevertheless, continuous exposure to high concentrations of this metal, mainly by inhalation (Dorman et al. 2006a), may produce alterations in the central nervous system.

Few studies have examined the effect of Mn on neurobehavioral functions in nonoccupationally exposed populations. Those studies did not report evident clinical effects; however, this does not preclude the existence of important cognitive deficits identified by detailed neuropsychological assessment (Mergler et al. 1999; Rodríguez-Agudelo et al. 2006).

Children are considered a vulnerable population, prone to cognitive alterations and long-term neurodevelopmental impairment due to neurotoxic agents (Erikson et al. 2007; Grandjean and Landrigan 2006; Weiss 2000). Lead (Pb), for example, is a confirmed neurotoxicant, and its detrimental effect on IQ has been the outcome most often studied (Needleman 2006; Weiss 2000). Because it is known that Mn exposure mainly affects the basal ganglia (Reaney et al. 2006) and frontal cortex (Schneider et al. 2009), cognitive and intellectual functions in children may be affected. A cross-sectional study performed in China demonstrated that 11- to 13-year-old children exposed to drinking water containing 0.24–0.36 mg/L Mn scored significantly lower in tests that evaluate manual skills, attention span, visual memory, and tracking than did a control group of children exposed to water with 0.03–0.04 mg/L Mn (He et al. 1994); most of the test scores correlated negatively with hair Mn (MnH). Wasserman et al. (2006) reported the results of a cross-sectional study on intellectual function in 10-year-old children exposed to Mn from drinking water in Bangladesh. Water Mn was associated with reduced Total, Verbal, and Performance IQ raw scores in a dose–response manner. Wasserman et al. (2008) then confirmed the negative associations with Mn exposure and added stunting as a meaningful variable associated with lower intellectual function.

Airborne Mn is considered the most hazardous route of exposure because Mn particles can enter into the body through the lung (Vitarella et al. 2000) and may access the brain directly through olfactory uptake (Tjälve and Henriksson 1999), bypassing homeostatic excretory mechanisms (Dorman et al. 2002). Children’s exposure to airborne Mn may be proportionally higher than that of adults. Not only are children exposed through breathing, but there can also be an additional risk from Mn-contaminated soil due to the children’s typical hand-to-mouth exposure (Aschner et al. 2007; Weiss 2000). To our knowledge, no study to date has examined the possible consequences of airborne environmental exposure to Mn on children’s intellectual function.

Previous studies in the Mexican mining district of Molango found that environmental exposure by inhaling Mn-rich dust was related to motor and attention impairments in adults from this population (Rodríguez-Agudelo et al. 2006; Solís-Vivanco et al. 2009). The objective of this cross-sectional study was to measure the environmental exposure to airborne Mn resulting from mining and processing and to explore its association with intellectual function of school-age children.

## Materials and Methods

### Subjects and study design

The present study is part of a larger project whose main objective is to determine the environmental and anthropogenic factors that explain the exposure to Mn in the mining district of Molango, as well as to design and carry out a risk management plan. The description of Mn sources and pathways includes spatial analysis of dust deposition and analysis of Mn concentration in particles. MiniVol samplers (Airmetrics Ltd., Eugene, OR, USA) with flows of 5 L/min using 47-mm Teflon filters (Whatman, Hillsboro, OR, USA) were used to monitor outdoor particulate matter ≤ 10 μm in aerodynamic diameter (PM_10_). The filters were analyzed to obtain the concentrations of Mn by particle-induced X-ray emission technique [see Supplemental Material (doi:10.1289/ehp.0901229)].

A cross-sectional study comparing exposed and nonexposed children was performed in Hidalgo State in central Mexico during 2006–2007. The Molango mining district has one of the largest Mn deposits around the world: It covers an area of 125 km^2^ and has a proven reserve of 32 million tons of Mn. Children from the closest rural communities (Tolago and Chiconcoac), located < 1 km from the Mn mineral processing plant, were considered the exposed population. Children included as control group were invited to participate from four rural communities in the Agua Blanca district: Chichicaxtle, El Palizar, Los Cubes, and Plan Grande. The Agua Blanca district is located 80 km southeast from the Mn source. These communities were selected because they showed socioeconomic conditions similar to those of the exposed group, according to the marginalization index assigned by the Mexican National Council of Population [Consejo Nacional de Población de México (CONAPO) 2005]. The selection criteria for the study were 7- to 11-year-old children (boys and girls) attending elementary school who had been living in the community for at least 5 years.

Children and parents were invited to participate voluntarily in this study by means of informative sessions and meetings held in the schools and health centers. The children who fulfilled the selection criteria were given an appointment at the health center of each community. Children and one of their parents gave informed consent to participate.

One hundred resident children from communities in the mining area were recruited as the exposed group, and 95 children from nonexposed communities were recruited as the control group. Both groups attended elementary school at the same school level. Children with a previous diagnosis of neurological or psychiatric disorders or with physical problems were excluded. The project was approved by bioethical committees of both the Instituto Nacional de Neurología y Neurocirugía “Manuel Velasco Suárez” (INNNMVS) and the Instituto Nacional de Salud Pública de México. The study complied with all applicable requirements according to the Declaration of Helsinki (World Medical Association 2002).

### Data collection

#### Questionnaires and assessment of intellectual function

Children’s mothers were asked to answer a sociodemographic questionnaire. Because the mother’s intellectual status is a known determinant of a child’s performance (Mink et al. 2004), the Progressive Matrices of the Raven test (Raven 1960) was applied to mothers to assess their intellectual function.

The revised version of the Wechsler Intelligence Scale for Children (WISC-R) adapted for Mexican population (Wechsler 1983) was applied to all of the children. WISC-R is the most frequently used psychometric test for the evaluation of children’s intellectual function in neurotoxicity studies (Walker et al. 2007) and is widely accepted in the scientific literature (Wechsler 1983, 1991). It consists of a battery of tests for 6- to 16-year-old children that evaluate intellectual abilities, and several scores are obtained. Verbal and Performance IQ scores are assessed separately, along with Total IQ score, which is an index of general intellectual functioning. The IQ scores were normalized by age based on a Mexican urban reference population (Wechsler 1983).

The WISC-R was applied in one session of approximately 45 min by two trained neuropsychologists from INNNMVS with a previously standardized administration method.

Along with the WISC-R, a neuropsychological test battery assessing verbal and visual memory, motor function, olfactory and vision function, and attention deficit/hyperactive and mood disorders was applied. Results from these tests will be published elsewhere.

#### Anthropometric measurements

Children’s height and weight were registered. These measurements, along with age, were used to obtain the anthropometric indices: weight for age, height for age, and body mass index (BMI) for age (de Onis et al. 2007). These indices were expressed in terms of *z*-scores. Stunting was defined as a height-for-age *z*-score < −2, wasting as a BMI-for-age *z*-score < −2, and overweight as a BMI-for-age *z*-score > 2 (World Health Organization 2006b).

#### Blood and hair sampling

Blood and hair samples were taken in the same session, 1 week after the cognitive evaluation. Samples (5 mL) were taken from cubital venous blood in metal-free Vacutainer EDTA tubes. All the samples were maintained refrigerated until analysis. Hair samples of about 0.5 g were taken from the occipital region, as close as possible to the scalp, and were stored in pretreated plastic bags until analysis. The blood and hair analyses were accomplished with samples of exposed and nonexposed individuals in the same session.

#### Blood measurements

Mn and Pb determinations were carried out with an atomic absorption spectrophotometer (model 3110; Perkin Elmer, Mexico City, Mexico), equipped with HGA-600 graphite furnace and AS-60 autosampler as previously described (Montes et al. 2008). Blood samples were also analyzed to determine hemoglobin (Hb) by routine procedure in the hospital facilities.

#### Hair measurements

Hair samples were washed three times by vigorous agitation with a detergent solution of 2% Triton X-100 and rinsed with deionized water. Samples were then dried at 60°C and cut into small pieces to facilitate acid digestion. Hair (300 mg) was placed in a metal-free polythene tube, and 250 μL of concentrated nitric acid was added (Suprapur; Merck, Naucalpan de Juárez, Estado de Mexico, Mexico). The samples were then digested for 30 min at 60°C. The resulting clear solution was analyzed by an atomic absorption spectrophotometer (Bouchard et al. 2007; Menezes-Filho et al. 2009). Quality control of the analysis of blood Mn (MnB) and MnH was assured by measuring a biological matrix–based reference material (bovine liver 1577b; National Institute of Standards and Technology, Gaithersburg, MD, USA) along with samples.

### Statistical analyses

Differences between groups were evaluated with the Mann–Whitney test for continuous or discrete variables and chi-square test for categorical variables. Total, Verbal, and Performance IQ were considered as the dependent variables (outcomes). Simple linear regressions were performed to assess the association of MnH and MnB with the outcome variables. We also performed simple linear regressions to assess the relation of blood Pb (PbB) and other potential covariates with the different outcomes. Exposure variables and other covariates significantly related to the outcomes and/or considered to be biologically relevant to the association were included in multiple regression models: PbB, age, sex, nutritional status, and maternal education and intelligence. The following variables were used as proxy measures of nutritional status: BMI-for-age *z*-scores, height-for-age *z*-scores, stunting (present or absent), and Hb level.

The product terms of the Mn exposure variables with PbB, age, sex, and nutritional status variables were also explored for possible heterogeneity effect between subgroups. Point estimates and confidence intervals (CIs) for the linear combinations of coefficients were computed after multiple regression with the significant product terms. Graphs were constructed to illustrate the effect modification by age and sex. Residual diagnostics were performed to assess the linear model assumptions and the presence of influential observations. A significance level of 0.05 was specified. Product (interaction) terms were retained in models if *p* < 0.20. All the analyses were performed with the statistical software Stata version 9.2 (StataCorp., College Station, TX, USA).

## Results

### Characteristics of the study population

Some participants refused to provide blood and hair samples (21 of the 100 exposed children, and 2 of the 95 control children). Thus, we carried out complete analyses for 172 children. Mothers who refused to provide biological samples showed significantly fewer years of education than did the other mothers. However, we found no other significant differences in sociodemographic characteristics or children’s IQ scores between participants with and without samples.

Table 1 shows the characteristics of the study population. The children in the exposed group were slightly older, fewer were overweight, and they presented a lower Hb concentration (*p* < 0.05) than did the control group. Stunting was more prevalent in the exposed group (21%) than in the control group (12%), although the difference was not statistically significant. Mothers in the exposed group showed lower performance in the Raven test. In the exposed group, 20% of fathers were miners; in the control group only 1% had this occupation. Other reported occupations were farming, construction, commerce, transport, and handicraft. We found no significant differences between groups regarding schooling or alcohol consumption by either parent. All the children had been living in the community of residence at least 5 years before the beginning of the study, and almost all (85%) were born and raised in the same community.

The 24-hr median Mn in PM_10_ in the exposed communities (0.13 μg/m^3^) was higher than in the control communities (0.02 μg/m^3^). The highest and lowest levels found in the exposed communities were 2.2 and 0.01 μg/m^3^, respectively.

### Biomarkers of exposure

The concentrations of MnB and MnH showed a low but significant correlation (*r* = 0.22, *p* < 0.01). Geometric mean MnB and MnH concentrations from the exposed group were significantly higher than those from the control group, and the range was also wider [Table 2; see also Supplemental Figure 1 (doi:10.1289/ehp.0901229)]. The median MnH concentration was almost 20 times higher in the exposed group (12.6 μg/g) than in the control group (0.6 μg/g). We found no significant sex differences for MnB (median, exposed vs. control: female, 9.5 vs. 8.5 μg/L; male, 9.7 vs. 8.0 μg/L) or MnH (median, exposed vs. control: female, 10.8 vs. 0.6 μg/g; male, 14.1 vs. 0.5 μg/g). MnH increased significantly with age [simple linear regression of age (in years) on the natural logarithm of MnH, for the population as a whole, β = 0.23; 95% CI, 0.06 to 0.40]. Age was not significantly associated with ln(MnB) in the population as a whole. Control children had higher PbB levels than did exposed children (Table 2). PbB was inversely correlated with MnB (*r* = −0.24, *p* < 0.01) and MnH (*r* = −0.60, *p* < 0.01) in the population as a whole. PbB was not significantly associated with age or sex.

### Intellectual function

Table 3 shows the results for the WISC-R, indicating that mean IQ levels in both exposure groups were below expected levels (90–110 IQ) (Wechsler 1983). Using the usual cutoff point published for this test, the total IQ was below average (< 90) for 82% in the total sample; this prevalence was significantly higher for the exposed group (92%) than for the control group (73%). The exposed group showed significantly lower Total, Verbal, and Performance IQ (75.1, 79.4, and 74.4, respectively) compared with those from the control group (82.2, 87.3, and 79.6, respectively). We observed the same trend for most of the subtests, especially for those that make up the Verbal IQ. In general, boys showed higher IQ scores compared with girls, and the younger children scored significantly higher than did the older ones (mean Total IQ: younger girls, 80.2 ; girls ≥ 9.5 years of age, 74.5; younger boys, 82.9; boys ≥ 9.5 years of age, 78.2).

### Association between Mn biomarkers and intellectual function

Hb levels were positively correlated with height-for-age *z*-scores (*r* = 0.31, *p* < 0.001) and BMI-for-age *z*-scores (*r* = 0.27, *p* < 0.001). We adjusted the final models for Hb, a variable that can be used as a proxy for nutritional and for iron status in children. The maternal scores on the Raven scale showed a significant correlation with years of education (*r* = 0.54, *p* < 0.001). This last variable had a stronger association with the outcome variables, and we included it in the final models. In this study, PbB was not significantly associated with IQ. In the control group, where > 25% of the children had PbB > 10 μg/dL, a stratified simple regression analysis indicated nonsignificant inverse associations with total IQ [β = −0.39; 95% confidence interval (CI), −1.25 to 0.47] and performance IQ (β = −0.68; 95% CI, −1.57 to 0.21) but not Verbal IQ (β = −0.02; 95% CI, −0.85 to 0.81). Results were similar after adjusting for covariates. Given prior evidence of the impact of this neurotoxicant on cognitive function, we included PbB in the final models to estimate effects of Mn biomarkers on IQ scores. Table 4 shows the results of unadjusted and adjusted linear regression models for the three outcomes and the two biomarkers of Mn exposure. MnH and MnB were inversely associated with Total IQ and Verbal IQ scores before adjusting for covariates. We also found a weak inverse association between MnH and Performance IQ.

**Figure 1:**
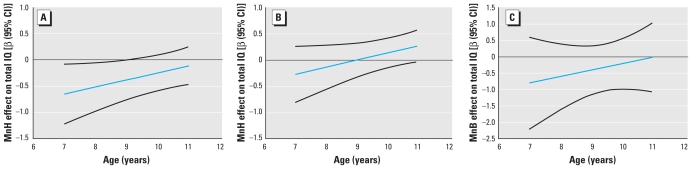
Modification by age and sex of the association between Mn exposure and Total IQ: association between MnH and Total IQ by age for girls (*A*) and for boys (*B*) and between MnB and Total IQ by age for all (*C*). Graphs were constructed with the computed point estimates and CIs (black lines) for the linear combinations of coefficients after the multiple regression with the significant product terms (results in Table 4).

Product terms between MnH and age (in years) and sex met our *a priori* criteria for effect modification (both *p* = 0.06), so we retained them in models for total IQ that we also adjusted for Hb (milligrams per deceliter) and maternal education (years). Figure 1 illustrates the effect modification of the association between MnH and total IQ by age and sex. These results indicate that the association was strongest in younger girls and weaker in the oldest girls in the study population (Figure 1A). In addition, we found little evidence of an association between MnH and total IQ among boys at any age (Figure 1B). We observed a similar pattern of associations between MnH and Verbal and Performance IQ scores [see Supplemental Material, Figures 2,3 (doi:10.1289/ehp.0901229)], with the strongest associations estimated for the youngest girls, and limited evidence of associations in boys at any age.

The results for MnB were in the same inverse direction but not significant, and we found no effect modification by sex (*p* > 0.90). Therefore, final models included the interaction with age and lower order terms for age, sex, Hb, and maternal education. The association between MnB and total IQ was also more pronounced at younger ages (in this case, for boys and girls combined), with little evidence of an association in older children (Figure 1C). We observed a similar pattern of associations between MnB and Verbal and Performance IQ scores [see Supplemental Material, Figures 2,3 (doi:10.1289/ehp.0901229)].

## Discussion

The main findings from this study suggest that MnH, a biomarker of chronic airborne environmental exposure, is inversely associated with intellectual function in young school-age children. The association was significantly modified by age and sex, with the strongest inverse associations in young girls, weaker inverse associations in older girls, and little evidence of associations in boys at any age. Associations with MnB did not appear to be modified by sex and appeared to be limited to younger study participants. Previous studies in the Molango mining district have shown airborne Mn exposure levels (median, 0.10 μg/m^3^) exceeding the recommended ones by the U.S. Environmental Protection Agency (1984) (0.05 μg/m^3^) (Rodríguez-Agudelo et al. 2006; Solís-Vivanco et al. 2009). Similarly, in this study we found a median airborne Mn level of 0.13 μg/m^3^. These high concentrations of airborne Mn were reflected in higher levels of Mn in biological samples from children living in the Molango mining district compared with other children. This is the first study assessing neurocognitive function in children exposed to airborne Mn.

Both exposed and nonexposed groups showed an average IQ lower than expected. However, these data can be considered an indicator of cultural bias present in any psychometric or neuropsychological test, including the intelligence scales (Ostrosky-Solís et al. 1985; Schettler 2001). Significant differences in the WISC-R scores between children who resided in urban and rural areas have been reported in Latin-American studies (Herrans and Rodríguez 1992). In these studies, the importance of residence as a main variable in WISC performance is described: Children of higher socioeconomic status showed better performance. Therefore, the low performance of both groups in our study is probably determined by prevailing socioeconomic conditions. However, this does not negate the evidence of an adverse effect of Mn exposure on IQ scores in younger children in the study population. This is the first report using the WISC-R test for Mexican rural (rather than urban) children. A study performed in Torreon (city located at the northern state of Coahuila) used only some subscales from WISC-R to evaluate urban children exposed to arsenic, and the results for the normalized scores were as follows: Arithmetic subscale, 7.4 ± 3.6; Digit Span subscale, 9.1 ± 3.6; Coding subscale, 2.3 ± 0.6 (Rosado et al. 2007). Compared with that study, our results showed a higher result for Arithmetic and Coding subscales but a lower result for Digit Span subscale. Disadvantaged children (because of poverty or low nutritional status, as in the case of these children) may show IQs around −2 SD (IQ = 50–69) (Grantham-McGregor et al. 2007; World Health Organization 2006a). A study that estimated the impact of metal exposure on intellectual function in a disadvantaged sample of Greek children reported a mean of 87 for Total IQ (Hatzakis et al. 1989). In other studies with metal-exposed children in developed countries (United States or China) but in disadvantaged conditions, Total IQ means of 87 (Dietrich et al. 1993) and 89 (Wang et al. 1989) were found.

The exposed group showed significantly higher levels of MnB and MnH than the control group. MnH has been used previously in studies of Mn environmental exposure. The exposed children in this study showed higher MnH concentrations than those reported by others. He et al. (1994) found mean levels of 1.25 μg/g in children exposed to Mn by drinking water; Wright et al. (2006) reported a mean MnH of 471.5 ppb (0.47 μg/g) in children living near a hazardous waste site. Higher concentrations of MnH (mean, 6.2 μg/g) have been reported in children exposed to Mn by drinking water (Bouchard et al. 2007), but these are lower than those found in the present study. Higher mean concentrations of MnH (15.2 μg/g) were reported in a recent study of children living in the vicinity of a ferromanganese alloy production plant in Brazil (Menezes-Filho et al. 2009).

MnH is controversial as a biomarker of exposure, with some groups suggesting that hair is not an appropriate matrix for estimating exposure to metals (Rodrigues and Batista 2008). One of the reasons is that the mechanism by which Mn is incorporated into hair is not clear; another reason is the uncertainty about the actual removal of external contamination by means of washing (Smolders et al. 2009). In the present study, we gave special care to washing and rinsing the sample before the metal analysis, in order to minimize external contamination. Our results show that exposed children had much higher MnH concentrations than did control subjects and provide information about a dose–response relationship with the studied neuropsychological outcome.

The association between MnH and intellectual function of children supports the results reported by Wright et al. (2006), who found an inverse relationship between the Total and Verbal IQ and MnH levels in children. Similar findings were reported for children exposed to high concentrations of Mn in drinking water (Wasserman et al. 2006, 2008). Mn exposure has been associated with other cognitive functions in children, such as attention, and with altered motor skills. Some studies have shown higher levels of MnH in children with attention deficits compared with controls (Barlow 1983; Bouchard et al. 2007).

The median MnB in exposed children (9.5 μg/L) was lower than that reported by Wasserman et al. (2006), who found a mean of 12.8 μg/L in the blood of children exposed to Mn by water intake. Another study (Röllin et al. 2005) reported MnB levels similar to those obtained in this study in South African children (9.8 μg/L) exposed to Mn from methylcyclopentadienyl manganese tricarbonyl, an octane-enhancing fuel additive. The present results were very similar to previous studies in adults from the same area that reported a mean for MnB of 9.7 μg/L (Montes et al. 2008) and 10.2 μg/L (Rodríguez-Agudelo et al. 2006). This suggests that the MnB levels are similar for adults and children who share sites of exposure to this metal.

We found an inverse but nonsignificant association between MnB and Total IQ. The relation between MnB and cognitive functions has been controversial. Some studies have found no association between MnB and IQ in children (Wasserman et al. 2006) or between MnB and motor tests in adults (Rodríguez-Agudelo et al. 2006), but others have found an association between MnB and deficient learning and memory performances (Mergler et al. 1999), Verbal IQ (Bowler et al. 2007), and Mini-Mental State Examination scores (Santos-Burgoa et al. 2001) for adult populations. Some authors have pointed out the difficulty in establishing MnB as a reliable biomarker of exposure (Mergler et al. 1999). Because of the distribution and relatively short half-life of Mn in the blood, it does not serve as a reliable indicator of the total body burden of Mn (Zheng et al. 2000). On an epidemiologic comparison basis, MnB levels may serve reasonably well as an indicator of recent Mn exposure. However, a relatively large variation in MnB among individuals, due to either diet or other unknown sources, suggests that this measure may be not the best parameter for the estimation of chronic exposure to this metal (Aschner et al. 2007; Montes et al. 2008).

Previous studies of adults have reported higher MnB concentrations in women than in men (Baldwin et al. 1999; Kondakis et al. 1989; Rodríguez-Agudelo et al. 2006), but we found no significant differences in MnB or MnH levels between boys and girls in our study population. However, estimated effects of MnH exposure on Verbal and Total IQ scores were strongest in younger girls, suggesting that this group could be more susceptible to effects of Mn on cognitive function. The mechanism underlying this association requires future research.

In adults, environmental Mn exposure has been associated with adverse effects on motor skills (Hudnell 1999; Mergler and Baldwin 1997; Mergler et al. 1999; Rodríguez-Agudelo et al. 2006) and attention (Solís-Vivanco et al. 2009), but our findings suggest effects on IQ in children. In particular, our results and those of others (Wasserman et al. 2006; Wright et al. 2006) suggest that the verbal processing functions may be most strongly affected.

Verbal comprehension, long- and short-term memory, and the competence to generate concepts are necessary functions for the integration of Verbal IQ (Sattler 1996; Wechsler 1991). It has been documented both in animal (Aschner et al. 2007; Shinotoh et al. 1995) and human (Huang et al. 2003; Shinotoh et al. 1997) studies that the neurotoxic effect and the accumulation of Mn in the adult nervous system is mainly located at the basal ganglia, as well as the frontal cortex (Dorman et al. 2006b; Schneider et al. 2009), and some cognitive functions related to frontal cortex and its connections with other cortical and subcortical areas could be affected by Mn (Schneider et al. 2006). It has also been reported that Mn affects the *N*-methyl-d-aspartate receptors, which are involved in learning (Guilarte and Chen 2007). The neurobiological mechanisms underlying the effects of Mn on the neurodevelopment or its relationship with intellectual processing in verbal domains in children have not been investigated; therefore, future studies are required to fully characterize these mechanisms.

Mothers whose children provided biological samples had a higher education level than did those who refused—even though they had agreed at the outset of their participation of the study. It is postulated that the results were not biased, because the IQ of the children without blood and hair samples was not significantly different from those who provided samples.

Although the control and exposure groups had similar socioeconomic and demographic characteristics, other characteristics may have differed. The control children had significantly higher PbB concentrations, and we included this variable in the final models to account for confounding due to effects of PbB on intellectual function.

The study design was cross-sectional, but the cognitive effects were not likely attributable to current or recent exposure. It has been shown that some factors in early infancy affect the cognitive outcome measured years later. For example, stunting during infancy has a strong adverse effect on cognitive function in late childhood (Berkman et al. 2002). A single exposure biomarker measurement might provide limited information about an individual’s exposure history, a difficulty regarding the interpretation of this cross-sectional study. Specifically, we measured PbB only once, and only after the period when children’s PbB levels usually peak (18–30 months) (Bellinger 2006). Recently, the age of greatest susceptibility to childhood Pb exposure has been discussed (Chen et al. 2005; Hornung et al. 2009). Several cohort studies have analyzed Pb-associated intellectual deficits, but there are no such longitudinal studies for Mn exposure in children. Age-related changes in vulnerability, and therefore the critical windows of exposure, remain uncertain.

Advances in research on exposure to Mn and its effects on the neurodevelopment become relevant because Mn is a trace essential element, and it is difficult to establish cutoff levels for adverse effects in children. A risk management program is being developed along with the residents, the mining company, and local authorities to propose strategies to diminish the exposure in the Molango mining area, as well as to develop educational remedial programs for the intellectual deficits of children living in this area.

## Figures and Tables

**Table 1:** Characteristics of the study population by group of exposure.

Characteristic	Control (*n* = 93)	Exposed (*n* = 79)
Children		
No. female	52	47
Age (years)	9.1 ± 1.5	9.8 ± 1.3*
Education (years)	3.1 ± 1.4	3.5 ± 1.3
Hb (g/dL)	14.1 ± 0.9	13.7 ± 0.8**
Anemia (Hb < 12 g/dL)	1	2
Stunting	12	21
Wasting	3	5
Overweight	12	1*

Families		
No. of children		
1–3	52	44
≥ 4	48	56
Mother’s age (years)	34.7 ± 6.0	35.0 ± 7.0
Raven raw scores	21.1 ± 6.5	16.7 ± 5.8#
Maternal education (years)	5.8 ± 3.2	4.9 ± 3.7
Married or living together	93	86
Housewife	89	92
Father’s occupation		
Miner	1	20#
Farmer	39	41
Construction worker	10	9
Others	50	30
Fuel for cooking purposes		
Firewood	36	40
Gas	11	19
Both	53	41

Values are percent or mean ± SD.

* *p* < 0.05

** *p* < 0.01, and

# *p* < 0.001 comparing exposed and control groups.

**Table 2:** Biomarkers of exposure: MnH, MnB, and PbB.

			Percentile of distribution					
Group	GM	95% CI	Min	50%	75%	90%	95%	Max
MnH (μg/g)								
Control	0.57	0.50–0.66	0.06	0.56	0.80	1.28	2.00	3.64
Exposed*	12.13	10.68–13.77	4.20	12.60	17.80	24.00	34.00	48.00

MnB (μg/L)								
Control	8.22	7.81–8.66	5.00	8.00	9.50	11.50	13.00	14.00
Exposed*	9.71	9.16–10.30	5.50	9.50	12.00	13.50	14.00	18.00

PbB (μg/dL)								
Control	7.96	7.32–8.64	1.85	8.00	10.50	12.50	15.50	22.50
Exposed*	3.37	2.91–3.90	0.50	3.30	5.50	7.50	8.50	13.50

Abbreviations: GM, geometric mean; Max, maximum; Min, minimum.

* *p* < 0.001 comparing exposed and control groups.

**Table 3:** WISC-R age-standardized scores by group of exposure (mean ± SD).

Measure	Control	Exposed
Total IQ	82.2 ± 14.7	75.1 ± 11.3*
Verbal IQ^a^	87.3 ± 14.1	79.4 ± 12.0*
Information	6.6 ± 2.9	5.4 ± 2.5#
Similarities	6.0 ± 3.7	6.1 ± 3.4
Arithmetic	10.2 ± 3.1	8.6 ± 2.8**
Vocabulary	9.2 ± 3.3	7.2 ± 3.3#
Comprehension	7.8 ± 2.5	6.2 ± 2.0#
Digit span	6.4 ± 2.8	5.4 ± 2.3**
Performance IQ^b^	79.6 ± 15.1	74.4 ± 13.0*
Picture completion	7.6 ± 3.1	6.6 ± 2.5**
Picture arrangement	6.5 ± 3.4	5.6 ± 2.9*
Block design	7.2 ± 3.1	6.7 ± 3.3
Object assembly	6.9 ± 3.1	6.0 ± 3.0
Coding	6.6 ± 3.1	5.9 ± 3.1
Mazes	10.4 ± 3.7	8.8 ± 3.4**

a Verbal IQ was calculated based on five subtests (information, similarities, arithmetic, vocabulary, and comprehension).

b Performance IQ was calculated based on five subtests (picture completion, picture arrangement, block design, object assembly, and coding).

* *p* < 0.05

** *p* < 0.01, and

# *p* < 0.001 comparing exposed and control groups.

**Table 4:** Simple and multiple linear regression models on IQ outcomes and Mn biomarkers [β (95% CI); *n* = 172 children].

Model	Total IQ	Verbal IQ	Performance IQ
Simple regression models for each of the biomarkers of exposure			
MnH (μg/g)	−0.20 (−0.42 to 0.02)	−0.29 (−0.51 to −0.08)	−0.08 (−0.32 to 0.16)
MnB (μg/L)	−0.40 (−1.19 to 0.39)	−0.69 (−1.48 to 0.09)	0.02 (−0.80 to 0.83)
PbB (μg/dL)	0.24 (−0.32 to 0.81)	0.45 (−0.10 to 1.01)	−0.02 (−0.63 to 0.60)

Multiple regression models for MnH with product terms			
MnH (μg/g)	−1.60 (−3.09 to −0.10)	−1.48 (−2.99 to 0.04)	−1.45 (−3.162 to 0.27)
PbB ≥ 6 μg/dL	−0.72 (−4.72 to 3.28)	−0.43 (−4.49 to 3.63)	−0.84 (−5.45 to 3.77)
Age (years)	−3.35 (−4.83 to −1.88)	−3.39 (−4.89 to −1.89)	−2.76 (−4.45 to −1.07)
MnH × age	0.14 (−0.01 to 0.28)	0.12 (−0.03 to 0.27)	0.12 (−0.04 to 0.29)
Male sex	1.51 (−2.94 to 5.96)	2.64 (−1.89 to 7.17)	−0.64 (−5.83 to 4.56)
MnH × sex	0.38 (−0.02 to 0.77)	0.28 (−0.12 to 0.68)	0.49 (0.04 to 0.95)
Hb (mg/dL)	2.77 (0.63 to 4.91)	2.71 (0.54 to 4.88)	2.36 (−0.11 to 4.84)
Maternal education (years)	0.97 (0.45 to 1.49)	0.85 (0.32 to 1.37)	0.92 (0.32 to 1.53)
* R*^2^	0.28	0.28	0.18

Multiple regression models for MnB with product terms^a^			
MnB (μg/L)	−2.18 (−6.93 to 2.57)	−3.50 (−8.13 to 1.13)	−2.29 (−7.58 to 2.99)
PbB ≥ 6 μg/dL	−1.02 (−4.68 to 2.65)	−0.80 (−4.38 to 2.78)	−1.02 (−5.09 to 3.04)
Age (years)	−4.45 (−9.15 to 0.25)	−5.79 (−10.37 to −1.20)	−4.30 (−9.53 to 0.94)
MnB × age	0.20 (−0.30 to 0.69)	0.31 (−0.18 to 0.79)	0.26 (−0.29 to 0.81)
Male sex	4.03 (0.51 to 7.56)	4.65 (1.20 to 8.10)	2.86 (−1.04 to 6.76)
Hb (mg/dL)	3.26 (1.13 to 5.39)	3.32 (1.25 to 5.40)	1.78 (−0.61 to 4.17)
Maternal education (years)	0.92 (0.39 to 1.44)	0.85 (0.34 to 1.37)	0.94 (0.36 to 1.52)
*R*^2^	0.25	0.29	0.15

a MnB × sex was not included because it was not significant with *p* < 0.20.
